# High-throughput nanoscale crystallization of di­hydro­pyridine active pharmaceutical ingredients

**DOI:** 10.1107/S2052520623010053

**Published:** 2023-12-21

**Authors:** Jessica P. Metherall, Philip A. Corner, James F. McCabe, Michael J. Hall, Michael R. Probert

**Affiliations:** a Chemistry, School of Natural and Environmental Sciences, Newcastle University, Newcastle upon Tyne, United Kingdom; b Early Product Development & Manufacturing, Pharmaceutical Sciences, BioPharmaceuticals R&D, AstraZeneca, Macclesfield, United Kingdom; IISER Kolkata, India

**Keywords:** di­hydro­pyridines, high-throughput crystallization, single-crystal X-ray diffraction, small molecule, encapsulated nanodroplet crystallization

## Abstract

Single-crystal X-ray diffraction analysis of small molecule active pharmaceutical ingredients is a key technique in the confirmation of molecular connectivity, including absolute stereochemistry, as well as the solid-state form. However, accessing single crystals suitable for X-ray diffraction analysis of an active pharmaceutical ingredient can be experimentally laborious, especially considering the potential for multiple solid-state forms (solvates, hydrates and polymorphs). In recent years, methods for the exploration of experimental crystallization space of small molecules have undergone a ‘step-change’, resulting in new high-throughput techniques becoming available. Here, the application of high-throughput encapsulated nanodroplet crystallization to a series of six di­hydro­pyridines, calcium channel blockers used in the treatment of hypertension related diseases, is described. This approach allowed 288 individual crystallization experiments to be performed in parallel on each molecule, resulting in rapid access to crystals and subsequent crystal structures for all six di­hydro­pyridines, as well as revealing a new solvate polymorph of nifedipine (1,4-dioxane solvate) and the first known solvate of nimodipine (DMSO solvate). This work further demonstrates the power of modern high-throughput crystallization methods in the exploration of the solid-state landscape of active pharmaceutical ingredients to facilitate crystal form discovery and structural analysis by single-crystal X-ray diffraction.

## Abstract 2

The use of encapsulated nanodroplet crystallization, for high-throughput screening of a selection of di­hydro­pyridine active pharmaceutical ingredients, resulted in access to single component crystalline forms for all examples as well as the discovery of two novel solvates.

## 1. Introduction

Understanding the solid-state landscape of a small molecule active pharmaceutical ingredient (API) is an important step in the development of a new drug, not least because the kinetic solubility of different forms can impact the bioavailability of the molecule. The discovery of crystalline forms of an API, and the growth of high-quality single crystals of those forms for diffraction analysis, is highly beneficial not only for validating connectivity and stereochemistry, but also to provide insight into the solid-state properties of the compound. The in-depth examination of the experimental crystallization space of a small molecule API by classical methods is, however, highly labour-intensive, due to the large number of experimental variables that need to be explored (Jones, 1981 ▸; Van der Sluis *et al.*, 1989 ▸; Spingler *et al.*, 2012 ▸; Wen *et al.*, 2019 ▸). Even with increasing automation, such studies are costly in both time and in the mass of sample required, and can still fail to identify important crystalline forms (*e.g.* polymorphs, solvates or hydrates) (Morissette *et al.*, 2004 ▸). However, modern approaches to high-throughput crystallization of APIs for single-crystal X-ray diffraction (SCXRD) are becoming more widely available, including ion exchange and vapour diffusion methods, microbatch under-oil techniques and Encapsulated Nanodroplet Crystallization (ENaCt) (Metherall *et al.*, 2023 ▸; Nievergelt *et al.*, 2018 ▸; Babor *et al.*, 2019 ▸; Tyler *et al.*, 2020 ▸). ENaCt is a high-throughput technique for the crystallization of organic soluble small molecules, in which nanolitre scale droplets of organic solvent containing only a few micrograms of analyte are encapsulated in a larger oil droplet, and allowed to crystallize. Using liquid handling robotics, large numbers of ENaCt experiments can be set-up in parallel, using multiwell plate formats, allowing rapid screening of large volumes of the multi-dimensional experimental space. Successful ENaCt experiments provide single crystals suitable for SCXRD, with ENaCt being previously applied to the crystallization of *N*-heterocyclic carbenes, aromatic polyketides, a SARS-CoV-2 protease inhibitor and cannabidiol (Zhu *et al.*, 2022 ▸; Al Subeh *et al.*, 2022 ▸; Cooper *et al.*, 2022 ▸; Straker *et al.*, 2023 ▸).

**Figure 1:**
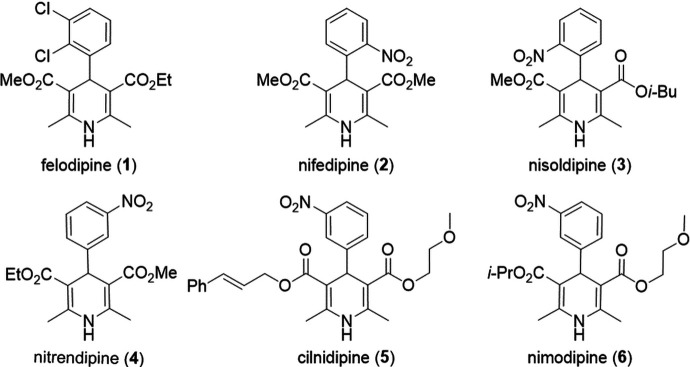
The six di­hydro­pyridine calcium channel blockers studied.

In this work we have examined ENaCt for the rapid parallel crystallization of a set pharmaceutically relevant APIs. Using a standardized approach, we postulated that crystal forms of a set of APIs could be accessed with minimal sample usage, a few milligrams, within two weeks for most molecules. We chose to study the di­hydro­pyridine calcium channel blockers, a widely used class of antihypertensive drugs, which form a chemically related series based around a core di­hydro­pyridine structure. The six di­hydro­pyridines investigated include felodipine (**1**) [(*rac*)-3-ethyl 5-methyl 4-(2,3-di­chloro­phenyl)-2,6-di­methyl-1,4-di­hydro­pyridine-3,5-di­carboxyl­ate, C_18_H_19_­Cl_2_NO_4_; CCDC reference 2263297], nifedipine (**2**) (di­methyl 2,6-di­methyl-4-(2-nitro­phenyl)-1,4-di­hydropyri­dine-3,5-di­carboxyl­ate, C_17_H_18_N_2_O_6_; CCDC reference 2263411, CCDC reference 2263278], nisoldipine (**3**) [(*rac*)-3-iso­butyl 5-methyl 2,6-di­methyl-4-(2-nitro­phenyl)-1,4-di­hydro­pyridine-3,5-di­carboxyl­ate, C_20_H_24_N_2_O_6_; CCDC reference 2298790], nitrendipine (**4**) [(*rac*)-3-ethyl 5-methyl 2,6-di­methyl-4-(3-nitro­phenyl)-1,4-di­hydro­pyridine-3,5-di­carboxyl­ate, C_18_H_20_N_2_O_6_; CCDC reference 2215879], cilnidipine (**5**) [(*rac*)-3-cinnamyl 5-(2-meth­oxy­ethyl) 2,6-di­methyl-4-(3-nitro­phenyl)-1,4-di­hydro­pyridine-3,5-di­carboxyl­ate, C_27_H_28_N_2_O_7_; CCDC reference 2215828] and nimodipine (**6**) [(*rac*)-3-iso­propyl 5-(2-meth­oxy­ethyl) 2,6-di­methyl-4-(3-nitro­phenyl)-1,4-di­hydro­pyridine-3,5-di­carboxyl­ate, C_21_H_26_N_2_O_7_; CCDC reference 2263295, CCDC reference 2215878] (Fig. 1 ▸).

## 2. Experimental

### 2.1. Materials

All compounds, oils, and solvents (Table S1, supporting information) were used as purchased without any further purification.

SWISSCI LCP glass plates with a 100 µm spacer and SWISSCI LCP cover glass slips were used as purchased.

### 2.2. Crystal growth by ENaCt

In order to develop a rapid, highly parallel, ENaCt screening method a range of four oils and 12 solvents were chosen, representing a variety of physical properties (Table S2).

The four different oils were selected to aid the encapsulation of the organic nanodroplets with variations in viscosity and miscibility with common organic solvents, including two fluorinated oils [Fomblin YR-1800 (FY) and Fluorinert FC-40 (FC-40)], a mineral oil (MO) and a silicone oil (PDMSO).

Di­methyl sulfoxide (DMSO), di­methyl formamide (DMF), methanol (MeOH), 2,2,2-tri­fluoro­ethanol (2,2,2-TFE), toluene, 1,2-di­chloro­ethane (DCE), 2-methyl­tetra­hydro­furan (2-MeTHF), 1,4-dioxane, ethyl acetate (EtOAc), aceto­nitrile (MeCN), 4-methyl-2-pentanone (MIBK) and nitro­methane (MeNO_2_) were among 12 different solvents selected for ENaCt experiments. They represent a range of solvent classes, boiling points, solubilizing power, solvent polarity and miscibility with the selected oils.

Stock solutions of each di­hydro­pyridine API were freshly prepared for each set of crystallization experiments. Samples were weighed (∼2 mg) into screw top vials and dissolved, through serial addition of solvent, to form a near-saturated solution (Table S3). This enabled preparation of samples near to the solubility limit on a small scale, without requiring knowledge of solubility information for each solute/solvent combination. Approximately 24 mg of di­hydro­pyridine API was employed in each case to prepare stock solutions, with around 0.25–0.5 µg used in each individual crystallization experiment. This approach allowed large numbers of experiments to be set up simultaneously with minimal sample requirements, in comparison to classical crystallization methods.

A 200 nL droplet of each oil was first deposited into the wells of a 96-well glass plate using an SPT Labtech mosquito liquid-handling robot (aspirate 1.0 mm min^−1^, dispense 1.0 mm min^−1^) (Fig. S1, supporting information). Each API stock solution (50 nL) was then injected into each oil droplet (aspirate 20 mm min^−1^, dispense 20 mm min^−1^). Plates were then sealed with a glass cover slip, checked by optical microscopy, and left for 14 days in the dark at ambient temperature (∼25°C).

For each di­hydro­pyridine API, three 96-well glass plates were employed, equating to 288 individual crystallization experiments including all combinations of the 12 solvents and four oils selected, as well as no-oil crystallization conditions (Fig. S1).

### 2.3. Characterization of crystallization outcomes

#### 2.3.1. Assessment of crystallization outcomes by optical microscopy

The crystallization plates were manually checked using optical microscopy for crystal growth. Observation of the experiment wells was carried out with a Nikon SMZ1000 microscope fitted with a linearly polarized light source and analyser. Digital images were captured with a GXCAM-U3-5 5.1 MP camera using the readily available *ToupView* (http://www.touptek.com) software. The crystallization outcomes were categorized as F = experimental failure, 1 = sample remaining in solution, 2 = non-crystalline, amorphous or oily material, 3 = microcrystalline material and 4 = single crystal(s) suitable for SCXRD analysis (Fig. S2).

#### 2.3.2. Single-crystal X-ray diffraction of crystals from ENaCt experiments

Upon observation of suitable crystals (grade 4), the relevant wells were opened with the use of a tungsten carbide scriber to remove a small portion of the glass cover slide, and the crystal manipulated using MiTeGen Kapton microtools. Crystals were transferred onto a standard MiTeGen Kapton loop and mounted onto an in-house diffractometer, or stored in a liquid N_2_ dry shipper for analysis on beamline I19 at Diamond Light Source, via remote access (Allan *et al.*, 2017 ▸; Johnson *et al.*, 2017 ▸). Unit-cell analysis of mounted crystals was undertaken, and full data collections were performed for all previously unknown di­hydro­pyridine API crystal forms, along with representative examples of known di­hydro­pyridine API crystal forms. In-house data were collected for **1** (form IV and form I), **2**, **2**·**1,4-dioxane**, **4**, **6** and **6**·**DMSO** using either a Bruker D8 Venture (**4** and **6**·**DMSO**) with IμS microfocus source (Cu *K*α1, λ = 1.54178 Å) and Photon II detector at 150 K, [data were reduced using *APEX3* software (Bruker, 2015 ▸), incorporating *SAINT* (v.8.40*B*; Bruker, 2017 ▸), *SADABS* was used for absorption correction (Bruker, 2016 ▸), or a Rigaku Oxford Diffraction Synergy-S diffractometer [**1** (form IV and form I), **2**, **2**·**1,4-dioxane** and **6**] (Cu *K*α, λ = 1.54184 Å) with a hybrid pixel array detector at 150 K [data were reduced using *CrysAlis PRO* with SCALE 3 ABSPACK correction implemented (Rigaku Oxford Diffraction, 2022 ▸)]. All samples were cooled, and temperature maintained using an Oxford Cryosystems Cryostream (Cosier & Glazer, 1986 ▸). Data for **3** and **5** were collected at Diamond Light Source at 100 K, using synchrotron radiation (λ = 1.54178 Å and λ = 0.6889 Å, respectively) and the data were processed using *APEX3* software. All structure solutions and refinements were completed using the *SHELX* suite (Sheldrick, 2015*a* ▸,*b* ▸) of programs via the *OLEX2* interface (Dolomanov *et al.*, 2009 ▸). For more details, see Tables 1 ▸ and 2 ▸.

Starting models from structure solution were completed and corrected through iterative rounds of least-squares minimization and analysis of Fourier difference maps of electron density. Felodipine (**1**, form IV) showed disorder in both side-chain terminal methyl groups. Nimodipine·DMSO (**6**·**DMSO**) showed disorder in the di­methyl sulfoxide solvent molecule (Table 2 ▸).

## 3. Results and discussion

### 3.1. Analysis of crystallization outcomes

**Figure 2:**
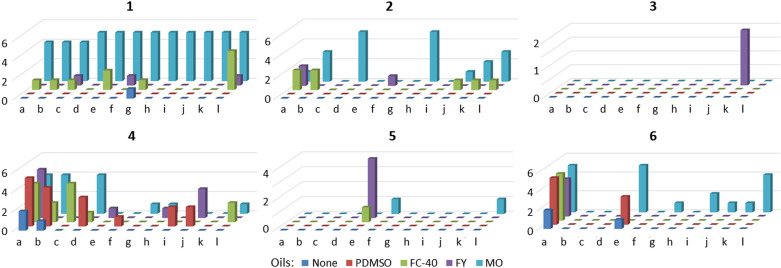
ENaCt solvent/oil combinations that provided crystals suitable for SCXRD analysis [grade 4 crystals (Fig. S2, supporting information)] for di­hydro­pyridines **1**–**6** y-axis: number of wells containing crystals of grade 4 and x-axis: solvent (a-l) used for the experiment [DMSO (*a*), DMF (*b*), MeOH (*c*), 2,2,2-TFE (*d*), toluene (*e*), DCE (*f*), 2-MeTHF (*g*), 1,4-dioxane (*h*), EtOAc (*i*), MeCN (*j*), MIBK (*k*) MeNO_2_ (l)].

The first crystals suitable for analysis by SCXRD were observed one hour following crystallization plate set-up, with further crystallization occurring and crystal growth over time. After 14 days no further significant change in the total number and size of crystals was observed and end-of-experiment plate readouts were performed (Section S5.2, supporting information) and crystallization outcomes recorded (Fig. 2 ▸).

For each di­hydro­pyridine API, the most successful solvent/oil combinations to produce crystals suitable for SCXRD analysis [grade 4 (Fig. S2)] were examined. Some molecules only provided crystalline material from a very limited set of conditions, such as a single solvent or oil, while others were more versatile, crystallizing from a wide variety of conditions. Across the di­hydro­pyridines examined (**1**–**6**), in all cases the use of an encapsulating oil was beneficial in comparison to the ‘no oil’ controls (Fig. 2 ▸).

The crystallization of felodipine (**1**) is clearly favoured in the presence of mineral oil, with every solvent in combination with mineral oil resulting in crystals suitable for SCXRD analysis. In this case the choice of oil had the biggest influence on the crystallization outcomes, with the solvent being less important, making felodipine (**1**) a partial outlier in the series, although MO also proved useful in the crystallization of nifedipine (**2**) and nimodipine (**6**). Interestingly, two polymorphs of felodipine (**1**) were identified, with form IV being obtained from DMSO, MeOH, toluene, DCE and MeNO_2_, whilst form I being obtained from DMF, 2,2,2-TFE, 2-MeTHF, 1,4-dioxane, EtOAc, MeCN and MIBK. The two crystals selected for structural determination were obtained from toluene/MO (form IV) (Fig. S11) and EtOAc/MO (form I) and correspond to the previously reported structures [CSD refcodes: DONTIJ03 (Surov *et al.*, 2012 ▸) and DONTIJ (Fossheim, 1986 ▸)].

Nifedipine (**2**), also gave a number of crystals of a suitable size and quality for analysis by SCXRD, again with mineral oil and FC-40 providing the most promising results, albeit with fewer overall hits than in the case of felodipine (**1**). The crystallization of nifedipine (**2**) appears to benefit from the use of more polar solvents including 2,2,2-TFE, MeNO_2_, DMF and DMSO, but also 1,4-dioxane. The crystal initially selected for SCXRD analysis came from 1,4-dioxane/MO (Fig. S11) and a nifedipine·1,4-dioxane solvate (**2**·**1,4-dioxane**) was obtained, which proved to be a new polymorph of the previously known nifedipine·1,4-dioxane solvate [CSD refcode: ASATOD (Caira *et al.*, 2003 ▸)]. A similarly high crystallization preference came from the use of 2,2,2-TFE as the solvent and, therefore, a crystal was also selected from 2,2,2-TFE/MO (Fig. S11). SCXRD analysis showed that the nifedipine (**2**) crystal matched the previously reported α form [CSD refcode: BICCIZ06 (Ou *et al.*, 2020 ▸)]. It should also be noted that unit-cell analysis also revealed the presence of the β form, although a full data collection was not possible from the crystals obtained. In the case of nifedipine (**2**), the α form is the more favoured polymorph, being obtained from DMSO, 2,2,2-TFE, 2Me-THF, MeCN, MIBK and MeNO_2_, whilst the β form was only observed from DMF and DCE.

The growth of single crystals of nisoldipine (**3**) suitable for analysis using SCXRD proved more challenging, only MIBK/FY proving successful and with no observable trends based on the appearance of grade 4 crystals (Fig. 2 ▸). However, if microcrystalline (grade 3) results are also taken into account, then a preference for toluene and MIBK as solvent can be observed (Fig. S9). The crystal selected and analysed using SCXRD came from MIBK/FY oil (Fig. S11) and corresponded to the previously reported structure [CSD refcode: FULPAD (Fossheim *et al.*, 1988 ▸)].

Nitrendipine (**4**) proved to be highly indiscriminate, providing crystals suitable for SCXRD across a wide range of different solvent and oil combinations, with DMSO and DMF being the most successful solvents. Even ‘no oil’ conditions gave good quality crystals from DMSO and DMF. The crystal selected and analysed by SCXRD came from DMF/MO (Fig. S11) and corresponded to the previously reported structure [CSD refcode: JEXKUS (Langs *et al.*, 1990 ▸)].

The crystallization of cilnidipine (**5**) by ENaCt proved more challenging, with toluene being the preferred solvent in combination with the fluorinated oils FC-40 and FY. Additionally, a small number of crystals were obtained from DCE and MeNO_2_, both in combination with mineral oil. The conditions used to grow the crystal analysed were toluene/FY (Fig. S11) and corresponded to the previously reported structure [CSD refcode: VELZUI (Hu *et al.*, 2006 ▸)].

Crystals of nimodipine (**6**) suitable for analysis were observed across multiple experimental conditions, showing a slight preference for the use of mineral oil for droplet encapsulation. The crystal initially selected was from MeNO_2_/MO (Fig. S11) and corresponded to the previously reported structure [CSD refcode: VAWWEW (Langs *et al.*, 1990 ▸)]. It was noteworthy that nimodipine (**6**) gave high-quality crystals from DMSO in combination with all four oils and even the no-oil conditions (Fig. 2 ▸). Since this strong solvent dependence was unusual, a crystal was selected from DMSO/FY (Fig. S11), resulting in the solution using SCXRD of a novel nimodipine·DMSO solvate (**6**·**DMSO**).

### 3.2. Analysis of the crystal structures

**Figure 3:**
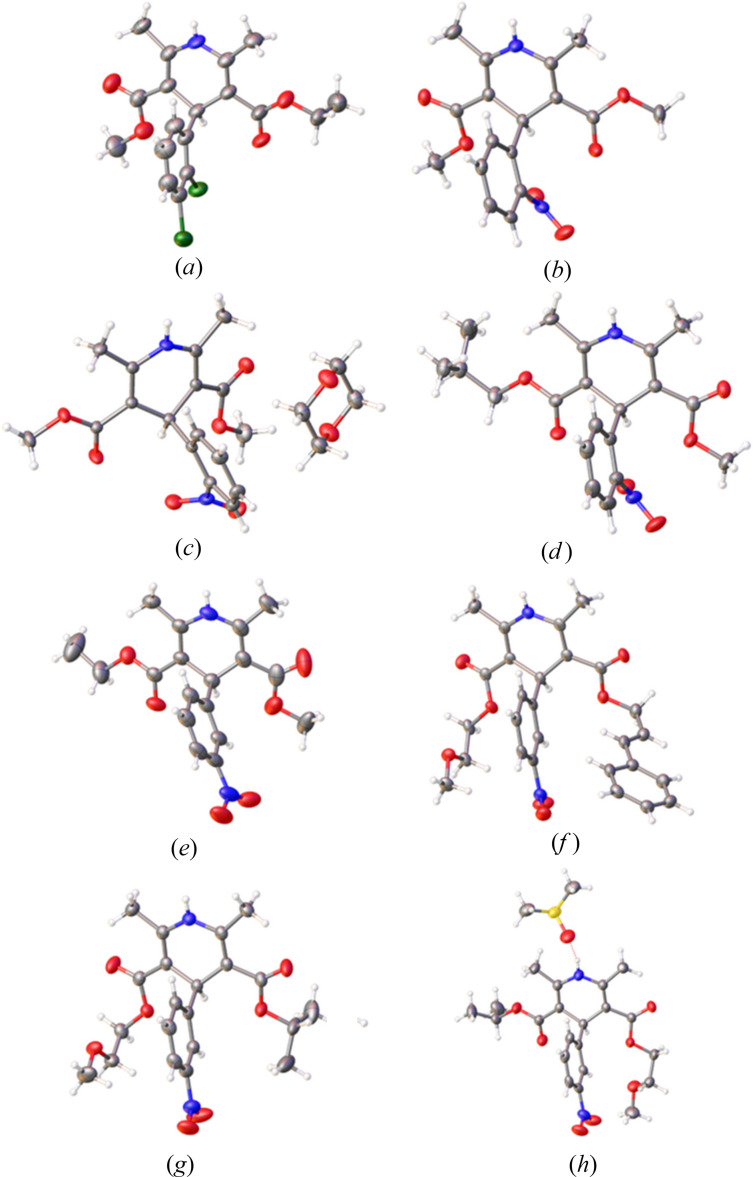
Displacement ellipsoid representation of (*a*) felodipine (form IV) (**1**), (*b*) nifedipine (α form) (**2**), (*c*) nifedipine·1,4-dioxane solvate (**2·1,4-dioxane**), (*d*) nisoldipine (**3**), (*e*) nitrendipine (**4**), (*f*) cilnidipine (**5**), (*g*) nimodipine (**6**) and (*h*) nimodipine·DMSO solvate (**6·DMSO**), with atomic displacement parameters drawn at the 50% probability level. Grey: carbon, blue: nitrogen, red: oxygen, green: chlorine and yellow: sulfur.

The crystal structures of di­hydro­pyridines **1–6**, including the novel solvates **2**·**1,4-dioxane** and **6**·**DMSO**, are now discussed in detail (Fig. 3 ▸).

For the non-solvated crystal structures (**1**–**6**), the structure similarity to published data is within error [CSD refcodes: (**1**) DONTIJ03 (form IV; Surov *et al.*, 2012 ▸), (**2**) BICCIZ06 (α form; Ou *et al.*, 2020 ▸), (**3**) FULPAD (Fossheim *et al.*, 1988 ▸), (**4**) JEXKUS (Langs *et al.*, 1990 ▸), (**5**) VELZUI (Hu *et al.*, 2006 ▸) and (**6**) VAWWEW (Langs *et al.*, 1990 ▸)]. For nifedipine (**2**), a novel monoclinic 1,4-dioxane solvate polymorph (**2**·**1,4-dioxane**) was obtained. This differs from the triclinic 1,4-dioxane solvate previously reported [CSD refcode: ASATOD (Caira *et al.*, 2003 ▸)]. The first reported solvate of nimodipine (**6**) was also discovered, in which nimodipine crystallized as a 1:1 DMSO solvate (**6**·**DMSO**).

### 3.3. Supramolecular features

**Figure 4:**
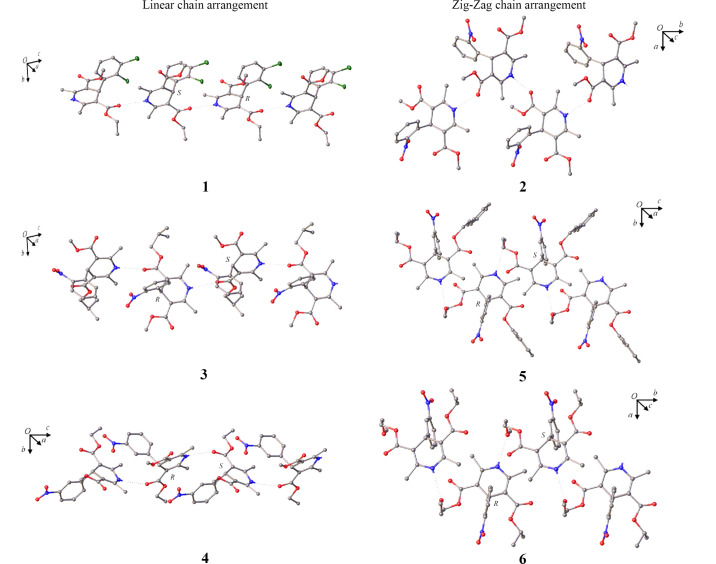
Supramolecular features of non-solvated di­hydro­pyridines **1**–**6** showing linear and zigzag chain arrangements. Extension of the hydrogen-bond network of **1** and **3** in the crystallographic [101] direction, **4** in the [001] direction, **2** in the [010] direction, **5** in the [001] direction and **6** in the [010] direction. Hydrogen atoms not involved in hydrogen bonding are omitted for clarity. Grey: carbon, blue: nitrogen, red: oxygen and green: chlorine.

In this section we compare the packing across the series of di­hydro­pyridine crystal forms, including a detailed analysis of the new solvates nifedipine·1,4-dioxane (**2**·**1,4-dioxane**) and nimodipine·DMSO (**6**·**DMSO**). The packing of all the non-solvated crystal structures (**1**–**6**) is dominated by one-dimensional N—H⋯O hydrogen-bond chains. All compounds besides nifedipine (**2**) have chiral centres, and all compounds besides cilnidipine (**5**) crystallize in centrosymmetric space groups, with both *R* and *S* enantiomers present for the chiral molecules. Cilnidipine (**5**) crystallized in the non-centrosymmetric space group *Fdd2*. For crystal structures **1**, **3** and **4** a linear supramolecular chain is observed, involving the di­hydro­pyridine N—H and the carbonyl oxygen in the side chain of the adjacent molecule. In contrast to this, crystal structures **2**, **5** and **6** show zigzag supramolecular chains involving the di­hydro­pyridine N—H and either a carbonyl oxygen (**2**) or an ether oxygen (**5** and **6**) in the side chain of the adjacent molecule (Fig. 4 ▸).

On closer inspection, the linear chain arrangement exists when a carbonyl oxygen in the 3,5-position (that is involved in the hydrogen-bond network) faces in the opposite (*anti*) direction to the di­hydro­pyridine N—H group (*anti*-carbonyl). In contrast to this, the zigzag arrangement exists when the carbonyl oxygen points in the same (*syn*) direction as the di­hydro­pyridine N—H (*syn*-carbonyl).

**Figure 5:**
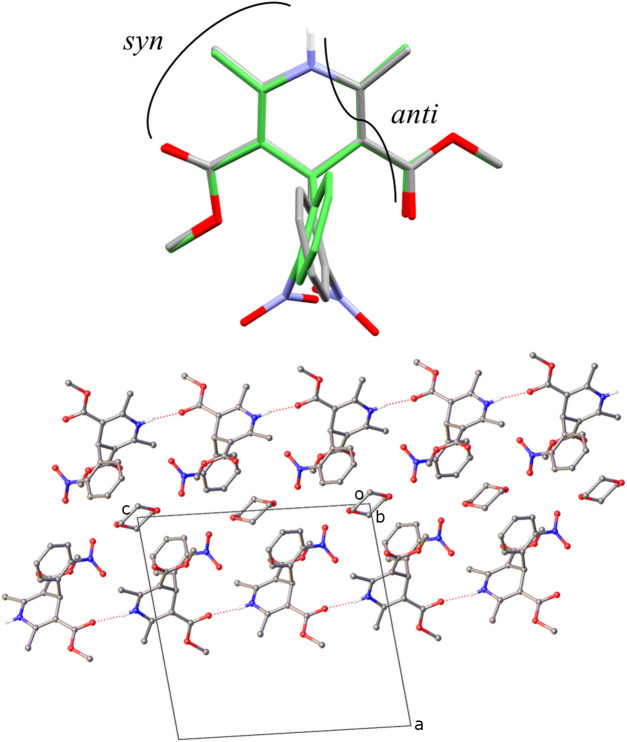
Top: overlay of nifedipine molecules from non-solvated (grey, **2**) and solvated (green, **2·1,4-dioxane**) crystal structures showing conformational similarity, *anti* and *syn* arrangements highlighted. Bottom: extension of the hydrogen-bond network viewed in the crystallographic (101) plane. Hydrogen atoms not involved in hydrogen bonding are omittedfor clarity.

The solvated crystal structure nifedipine·1,4-dioxane (**2**·**1,4-dioxane**) shows a linear supramolecular chain involving an *anti* configuration of the di­hydro­pyridine N—H and the carbonyl oxygen in the side chain of the adjacent molecule (Fig. 5 ▸). This is in contrast with the non-solvated crystal structure of nifedipine (**2**), in which a zigzag arrangement is observed.

The change in the packing of the solvate is due to the inclusion of the 1,4-dioxane solvent molecule. The 1,4-dioxane molecule occupies channels nearest to the *syn-*carbonyl oxygen, previously used in the extended hydrogen-bond network, thus only the *anti-*carbonyl oxygen is available for inclusion in the supramolecular chain.

**Figure 6:**
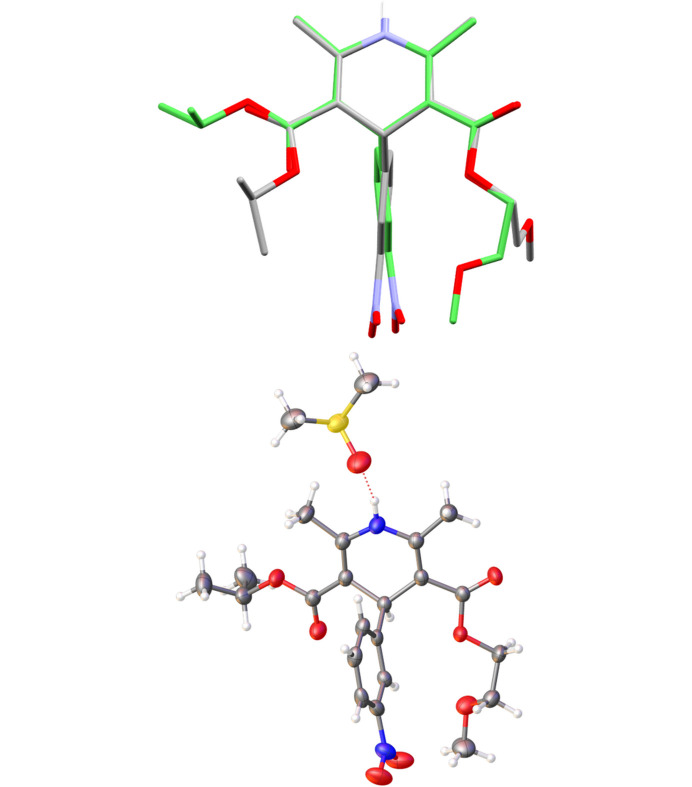
Top: overlay of nimodipine molecules from non-solvated (grey, **6**) and solvated (green, **6·DMSO**), hydrogen atoms not involved in hydrogen bonding are omitted for clarity. Bottom: finite hydrogen-bonded unit of **6·DMSO**. Grey: carbon, blue: nitrogen, red: oxygen and yellow: sulfur.

In the case of the nimodipine·DMSO solvate (**6**·**DMSO**), the inclusion of DMSO disrupts the common hydrogen-bond networks observed across the related di­hydro­pyridine structures, in this case disrupting the di­hydro­pyridine N—H⋯O (ether) hydrogen bond. Thus, the nimodipine·DMSO solvate (**6**·**DMSO**) crystallizes as a finite hydrogen-bonded unit, in which the DMSO hydrogen bonds to nimodipine via the di­hydro­pyridine N—H bond. The disruption of the hydrogen-bond network can also be seen through overlay of the molecules within the crystal structures of **6** and **6**·**DMSO**, which shows that the ether-containing side chain has reoriented from the non-solvated to the solvated structure (Fig. 6 ▸).

### 3.4. Hirshfeld surface analysis

Key features of intermolecular interactions in the crystal structures of the di­hydro­pyridine APIs can be more easily visualized with the aid of Hirshfeld surfaces.

**Figure 7:**
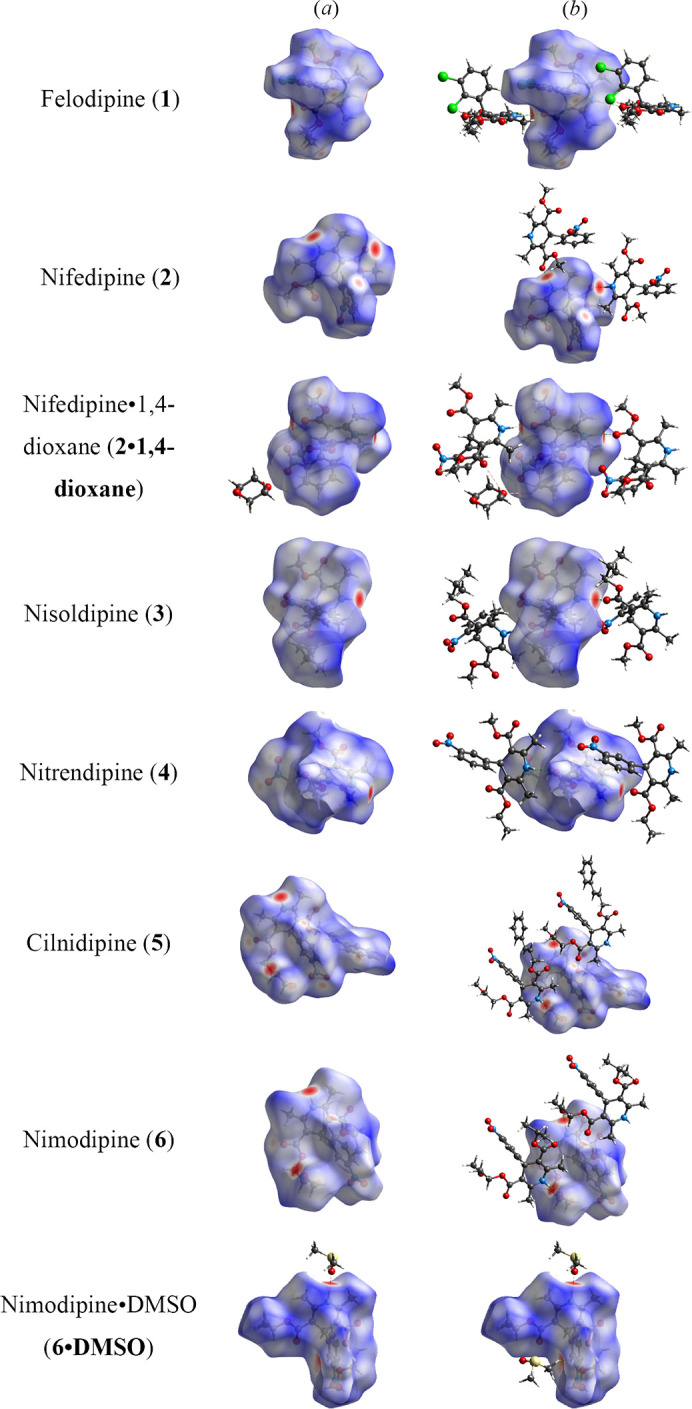
Hirshfeld *d*_norm_ (*a*) for crystal structures (**1**–**6**), including the novel solvates **2**·**1,4-dioxane** and **6·DMSO** and (*b*) intermolecular interactions between neighbouring molecules. Grey: carbon, blue: nitrogen, red: oxygen, green: chlorine and yellow: sulfur.

The molecular Hirshfeld surfaces, *d*_norm_ (normalised contact distance), were generated using *CrystalExplorer21* (Spackman *et al.*, 2021 ▸) for crystal structures **1**–**6**, including the novel solvates **2**·**1,4-dioxane** and **6**·**DMSO**. Strong hydrogen-bond interactions, such as O-H⋯N, are seen for all, depicted as a bright-red area on the Hirshfeld surface (Sen *et al.*, 2018 ▸) (Fig. 7 ▸).

**Figure 8:**
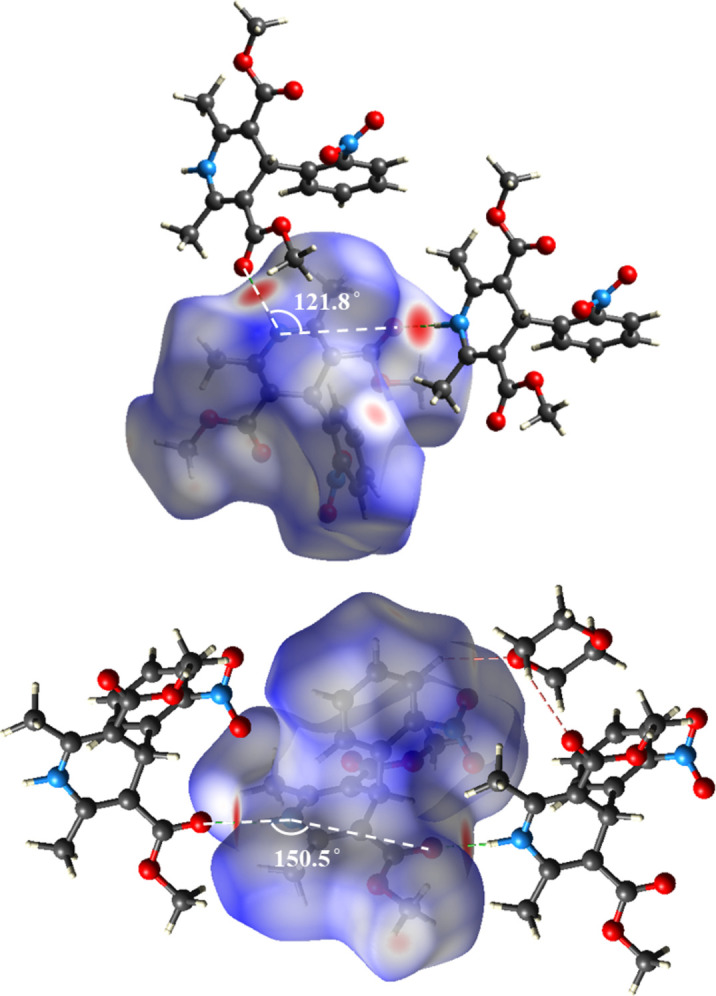
Hirshfeld surfaces for nifedipine (**2**) and nifedipine·1,4-dioxane solvate (**2·1,4-dioxane**) displaying the zigzag and linear arrangement of the hydrogen-bond network.

The differences between the zigzag arrangements (**2**, **5** and **6**) and linear arrangements (**1**, **3** and **4**) can be readily seen in the Hirshfeld surfaces, particularly in the case of nifedipine (**2**) and nifedipine·1,4-dioxane solvate (**2**·**1,4-dioxane**). In the structure of nifedipine (**2**), the zigzag hydrogen bonding results in an angle of 121.8° between the carbonyl oxygen on one molecule, the di­hydro­pyridine nitro­gen atom and the carbonyl oxygen on the adjacent molecule. In comparison, the linear hydrogen-bond network in nifedipine·1,4-dioxane solvate (**2**·**1,4-dioxane**) results in an angle of 150.5° between the carbonyl oxygen on one molecule, the di­hydro­pyridine nitro­gen atom and the carbonyl oxygen on the adjacent molecule (Fig. 8 ▸).

Across the series of the di­hydro­pyridine crystal structures studied, in almost all cases ‘hot spots’ corresponding to the hydrogen bonding interactions with either a carbonyl or an ether oxygen are present. The Hirsheld surface of nimodipine·DMSO solvate (**6**·**DMSO**) shows that this is the exception. The inclusion of DMSO into the structure interrupts the hydrogen bonding interactions with adjacent nimodipine molecules, such as seen in the Hirsheld surface of **6**. As a result, the ‘hot spot’ around the ether oxygen is no longer present in the Hirsheld surface of **6**·**DMSO**, the major interaction being the hydrogen bond from the di­hydro­pyridine N—H to the DMSO oxygen. The Hirsheld surface also highlights a weak inter­action with the DMSO hydrogen and the aromatic ring of nimodipine (Fig. 7 ▸).

## 4. Conclusions

Six di­hydro­pyridine APIs were successfully crystallized using the high-throughput parallel ENaCt method. A total of 1728 individual crystallization experiments were performed, 288 per compound covering 60 different experimental conditions (solvent/oil combinations), with minimal experimental set-up time. In all cases, single crystals suitable for SCXRD were obtained within two weeks. Analysis of the successful crystallization ‘hot spots’ from ENaCt allowed the detection of two previously unreported crystalline forms, a new polymorph of the nifedipine·1,4-dioxane solvate (**2**·**1,4-dioxane**) and the first known solvate of nimodipine, nimodipine·DMSO (**6**·**DMSO**). In addition, two polymorphs of both felodipine (**1**) and nifedipine (**2**) were observed. This rapid access to crystalline forms for a series of APIs, along with the discovery of the novel solvates, demonstrates the potential of high-throughput ENaCt screening methods for application to the discovery of API crystalline forms. It is also interesting to note, for some di­hydro­pyridines, the choice of solvent appeared to be the major factor in determining successful crystallization [*e.g.* cilnidipine (**5**)/toluene] whilst for others the oil employed was the major driving factor [*e.g.* felodipine (**1**)/MO]. Further research to better understand the role of solvents and oils in ENaCt for APIs is the subject of ongoing work.

## Supplementary Material

Crystal structure: contains datablock(s) global, felodipine, nifedipine, nifedipine-dioxane, nisoldipine, nitrendipine, cilnidipine, nimodipine, nimodipine-dmso. DOI: 10.1107/S2052520623010053/rm5073sup1.cif

Structure factors: contains datablock(s) felodipine. DOI: 10.1107/S2052520623010053/rm5073felodipinesup2.hkl

Structure factors: contains datablock(s) nifedipine. DOI: 10.1107/S2052520623010053/rm5073nifedipinesup3.hkl

Structure factors: contains datablock(s) nifedipine-dioxane. DOI: 10.1107/S2052520623010053/rm5073nifedipine-dioxanesup4.hkl

Structure factors: contains datablock(s) nisoldipine. DOI: 10.1107/S2052520623010053/rm5073nisoldipinesup5.hkl

Structure factors: contains datablock(s) nitrendipine. DOI: 10.1107/S2052520623010053/rm5073nitrendipinesup6.hkl

Structure factors: contains datablock(s) cilnidipine. DOI: 10.1107/S2052520623010053/rm5073cilnidipinesup7.hkl

Structure factors: contains datablock(s) nimodipine. DOI: 10.1107/S2052520623010053/rm5073nimodipinesup8.hkl

Structure factors: contains datablock(s) nimodipine-dmso. DOI: 10.1107/S2052520623010053/rm5073nimodipine-dmsosup9.hkl

Tables S1-S3, Figs. S1-S11. DOI: 10.1107/S2052520623010053/rm5073sup10.pdf

Supporting information file. DOI: 10.1107/S2052520623010053/rm5073nifedipinesup11.cml

Supporting information file. DOI: 10.1107/S2052520623010053/rm5073nifedipine-dioxanesup12.cml

CCDC references: 2215828, 2215878, 2215879, 2263278, 2263295, 2263297, 2263411, 2298790

## Figures and Tables

**Table 1:** Crystallographic data and corresponding refinement information for **1** (form IV), **2** (α form), **2**·**1,4-dioxane**) and **3** H atoms treated by a mixture of independent and constrained refinement for all structures.

	Felodipine (**1**, form IV)	Nifedipine (**2**, α form)	Nifedipine 1,4-dioxane (**2**·**1,4-dioxane**)	Nisoldipine (**3**)
Crystal data				
Chemical formula	C_18_H_19_Cl_2_NO_4_	C_17_H_18_N_2_O_6_	C_17_H_18_N_2_O_6_·C_2_H_4_O	C_20_H_24_N_2_O_6_
*M*_r_	384.24	346.33	390.38	388.41
Crystal system, space group	Monoclinic, *P*2_1_/*n*	Monoclinic, *P*2_1_/*c*	Monoclinic, *P*2_1_/*c*	Monoclinic, *P*2_1_/*n*
Temperature (K)	150	150	150	100
*a*, *b*, *c* (Å)	11.1017 (7), 12.5717 (7), 13.5023 (8)	10.6713 (3), 10.3971 (3), 14.7824 (3)	14.0927 (5), 9.2253 (4), 14.5139 (5)	10.7862 (1), 15.6320 (1), 11.9241 (1)
α, β, γ (°)	90, 107.056 (6), 90	90, 94.545 (2), 90	90, 97.164 (3), 90	90, 102.528 (1), 90
*V* (Å^3^)	1801.60 (19)	1634.96 (7)	1872.21 (12)	1962.65 (3)
*Z*	4	4	4	4
Radiation type, λ (Å)	Cu *K*α, 1.54184	Cu *K*α, 1.54184	Cu *K*α, 1.54184	Synchrotron, 1.0402
μ (mm^−1^)	3.44	0.91	0.90	0.81
Crystal size (mm)	0.45 × 0.32 × 0.29	0.51 × 0.45 × 0.22	0.52 × 0.47 × 0.33	0.21 × 0.03 × 0.02

Data collection				
Absorption correction	Multi-scan (*CrysAlis PRO*). Empirical absorption correction using spherical harmonics, implemented in SCALE3 ABSPACK scaling algorithm.	Multi-scan (*CrysAlis PRO*). Empirical absorption correction using spherical harmonics, implemented in SCALE3 ABSPACK scaling algorithm.	Multi-scan (*CrysAlis PRO*). Empirical absorption correction using spherical harmonics, implemented in SCALE3 ABSPACK scaling algorithm.	Multi-scan (*SADABS2016/2*).
*T*_min_, *T*_max_	0.738, 1.000	0.869, 1.000	0.909, 1.000	0.663, 0.753
No. of measured, independent and observed [*I* > 2σ(*I*)] reflections	10365, 3494, 2903	10751, 3255, 2857	11509, 3659, 3110	15426, 3749, 3554
*R*_int_	0.031	0.031	0.024	0.031
(sin θ/λ)_max_ (Å^−1^)	0.626	0.632	0.625	0.619

Refinement				
*R*[*F* ^2^ > 2σ(*F* ^2^)], *wR*(*F* ^2^), *S*	0.056, 0.160, 1.05	0.041, 0.119, 1.06	0.035, 0.095, 1.05	0.045, 0.126, 1.05
No. of reflections	3494	3255	3659	3749
No. of parameters	262	234	261	263
No. of restraints	204	0	198	210
Δρ_max_, Δρ_min_ (e Å^−3^)	0.41, −0.48	0.41, −0.18	0.23, −0.20	0.69, −0.21

Computer programs: *CrysAlis PRO* (v.1.171.42.73a; Rigaku OD, 2022 ▸), GDA 8.44 (generic data acquisition software), *APEX3* (v.2017.3-0; Bruker, 2017 ▸), *SADABS2016/2* (Bruker, 2016 ▸), *SAINT* (Bruker, 2017 ▸), *SHELXT 2018/2* (Sheldrick, 2015*a* ▸), *SHELXL 2018/3* (Sheldrick, 2015*b* ▸), *OLEX2* (v.1.5; Dolomanov *et al.*, 2009 ▸).

**Table 2:** Crystallographic data and corresponding refinement information for **4**, **5**, **6** and **6**·**DMSO** H atoms treated by a mixture of independent and constrained refinement for all structures.

	Nitrendipine (**4**)	Cilnidipine (**5**)	Nimodipine (**6**)	Nimodipine·DMSO (**6**·**DMSO**)
Crystal data				
Chemical formula	C_18_H_20_N_2_O_6_	C_27_H_28_N_2_O_7_	C_21_H_26_N_2_O_7_	C_2_H_6_OS·C_21_H_26_N_2_O_7_
*M*_r_	360.36	492.51	418.44	496.56
Crystal system, space group	Monoclinic, *P*2_1_/*c*	Orthorhombic, *F* *d* *d*2	Monoclinic, *P*2_1_/*c*	Triclinic, *P* 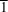
Temperature (K)	150	100	150	150
*a*, *b*, *c* (Å)	8.8143 (5), 15.3632 (8), 12.9602 (7)	15.0989 (17), 59.567 (7), 10.9540 (12)	13.8103 (2), 10.7631 (2), 14.8187 (3)	9.5050 (8), 11.865 (1), 12.7533 (10)
α, β, γ (°)	90, 93.615 (2), 90	90, 90, 90	90, 104.604 (2), 90	63.606 (2), 77.493 (2), 89.029 (2)
*V* (Å^3^)	1751.52 (16)	9852.0 (19)	2131.51 (7)	1252.59 (18)
*Z*	4	16	4	2
Radiation type, λ (Å)	Cu *K*α, 1.54184	Synchrotron, 0.6889	Cu *K*α, 1.54184	Cu *K*α, 1.54184
μ (mm^−1^)	0.87	0.09	0.82	1.57
Crystal size (mm)	0.56 × 0.43 × 0.12	0.56 × 0.14 × 0.03	0.55 × 0.52 × 0.07	0.45 × 0.34 × 0.09

Absorption correction	Multi-scan (*SADABS2016/2*). *wR*_2_(int) was 0.1443 before and 0.0747 after correction. The ratio of minimum to maximum transmission is 0.8812. The λ/2 correction factor is not present.	Multi-scan (*SADABS2016/2*). *wR*_2_(int) was 0.1842 before and 0.0893 after correction. The ratio of minimum to maximum transmission is 0.6128. The λ/2 correction factor is not present.	Multi-scan (*CrysAlis PRO*). Empirical absorption correction using spherical harmonics, implemented in SCALE3 ABSPACK scaling algorithm.	Multi-scan (*SADABS2016/2*). *wR*_2_(int) was 0.0786 before and 0.0589 after correction. The ratio of minimum to maximum transmission is 0.8437. The λ/2 correction factor is not present.
*T*_min_, *T*_max_	0.663, 0.753	0.457, 0.745	0.788, 1.000	0.635, 0.753
No. of measured, independent and observed [*I* > 2σ(*I*)] reflections	21238, 3107, 2820	15527, 4234, 3656	14528, 4200, 3488	19742, 4381, 4128
*R*_int_	0.043	0.057	0.030	0.033
(sin θ/λ)_max_ (Å^−1^)	0.596	0.597	0.633	0.596

Refinement				
*R*[*F* ^2^ > 2σ(*F* ^2^)], *wR*(*F* ^2^), *S*	0.057, 0.156, 1.05	0.040, 0.094, 0.99	0.042, 0.119, 1.05	0.050, 0.132, 1.03
No. of reflections	3107	4234	4200	4381
No. of parameters	243	339	280	394
No. of restraints	195	1	0	375
Δρ_max_, Δρ_min_ (e Å^−3^)	0.39, −0.27	0.19, −0.17	0.29, −0.24	0.45, −0.32
Absolute structure	–	Flack *x* determined using 1451 [(*I* ^+^) − (*I* ^−^)]/[(*I* ^+^) + (*I* ^−^)] (Parsons *et al.*, 2013 ▸).	–	–
Absolute structure parameter	–	−0.6 (7)	–	–

Computer programs: *CrysAlis PRO* (v.1.171.42.73a; Rigaku OD, 2022 ▸), GDA 8.44 (generic data acquisition software), *APEX3* (v.2017.3-0; Bruker, 2017 ▸), *SADABS2016/2* (Bruker, 2016 ▸), *SAINT* (Bruker, 2017 ▸), *SHELXT 2018/2* (Sheldrick, 2015*a* ▸), *SHELXL 2018/3* (Sheldrick, 2015*b* ▸), *OLEX2* (v.1.5; Dolomanov *et al.*, 2009 ▸).
