# Supplementary material for: High-throughput nanoscale crystallization of di­hydro­pyridine active pharmaceutical ingredients
Source: Acta Crystallogr B Struct Sci Cryst Eng Mater. 2023 Dec 21;80(Pt 1):4–12. doi: 10.1107/S2052520623010053 (PMC10848412; doi:10.1107/S2052520623010053)
Supplement: Supplementary file 10 [file b-80-00004-sup10.pdf]

STRUCTURAL SCIENCE
CRYSTAL ENGINEERING
MATERIALS

Volume 80 (2024)

Supporting information for article:

High-Throughput Nanoscale Crystallisation of Dihydropyridine Active Pharmaceutical Ingredients

Jessica P., Jessica P. Metherall, Philip A. Corner, James F. McCabe, Michael J. Hall and Michael R. Probert

S1. Abbreviations

Table S1 Abbreviations

ENaCt Oils	
PDMSO	Poly(dimethylsiloxane); CAS: 63148-62-9; supplier: Sigma Aldrich
FC-40	Fluorinert FC-40; CAS: 51142-49-5; supplier: Fluorochem
FY	Fomblin YR-1800; CAS: 69991-67-9; supplier: Alfa Aesar
MO	Mineral oil; CAS: 8042-47-5; supplier: Sigma Aldrich
ENaCt Solvents	
DMSO	Dimethyl sulfoxide
DMF	Dimethylformamide
MeOH	Methanol
2,2,2-TFE	2,2,2-Trifluoroethanol
Toluene	Toluene
DCE	1,2-Dichloroethane
2-MeTHF	2-Methyltetrahydrofuran
1,4-Dioxane	1,4-Dioxane
EtOAc	Ethyl acetate
MeCN	Acetonitrile
MIBK	4-Methylpentan-2-one
$MeNO_2$	Nitromethane

S2. Solvent Properties

Table S2 Solvent Properties

Solvent	Classification	Boiling Point (ºC)	Density (g mL^{-1})	Dielectric Constant (ε)
DMSO	Aprotic polar	189	1.092	47
DMF	Aprotic polar	153	0.945	38.3
MeOH	Alcohol	64.6	0.791	32.6
2,2,2-TFE*	Alcohol	73.6	1.38	-
Toluene	Hydrocarbon	110.6	0.867	2.4
DCE	Halogenated	83.5	1.245	10.4
2-MeTHF**	Ether	78	0.855	-
1,4-Dioxane	Ether	101.1	1.033	2.2
EtOAc	Ester	77	0.895	6
MeCN	Aprotic polar	81.7	0.786	36.6
MIBK***	Ketone	115.8	0.80	-
$MeNO_2$	Aprotic polar	101.2	1.382	35.9

All data apart from those indicated were reported in Lide, D. R. (2006). Handbook of Chemistry and Physics, Ed. 87, pp. 2608. Boca Raton: CRC Press. Classifications adapted from Prat, D., Wells, A., Hayler, J., Sneddon, H., McElroy, C. R., Abou-Shehada, S. & Dunn, P. J. (2016). *Green Chem.* **18**, 288–296.

* Data obtained from supplier site: https://www.merckmillipore.com/GB/en/product/222-Trifluoroethanol,MDA_CHEM-808259

** Data obtained from supplier site: https://www.merckmillipore.com/GB/en/product/2-Methyltetrahydrofuran,MDA_CHEM-821093

*** Data obtained from supplier site: https://www.sigmaaldrich.com/GB/en/sds/usp/1430203

S3. Sample Preparation

Table S3 Concentrations

	Vial	Solvent	Mass / mg	Volume / µL	Conc. / mg mL^{-1}
Felodipine (**1**)	1	Dimethyl sulfoxide (DMSO)	2	12	167
	2	Dimethylformamide (DMF)	2	12	167
	3	Methanol (MeOH)	2	24	83
	4	2,2,2-Trifluoroethanol (2,2,2-TFE)	2	24	83
	5	Toluene	2	48	42
	6	1,2-Dichloroethane (DCE)	2	12	167
	7	2-Methyltetrahydrofuran (2-MeTHF)	2	12	167
	8	1,4-Dioxane	2	24	83
	9	Ethyl acetate (EtOAc)	2	12	167
	10	Acetonitrile (MeCN)	2	12	167
	11	4-Methylpentan-2-one (MIBK)	2	24	83
	12	Nitromethane ($MeNO_2$)	2	48	42
Nifedipine (**2**)	13	Dimethyl sulfoxide (DMSO)	2	24	83
	14	Dimethylformamide (DMF)	2	24	83
	15	Methanol (MeOH)	2	96	21
	16	2,2,2-Trifluoroethanol (2,2,2-TFE)	2	24	83
	17	Toluene	2	96	21
	18	1,2-Dichloroethane (DCE)	2	48	42
	19	2-Methyltetrahydrofuran (2-MeTHF)	2	24	83
	20	1,4-Dioxane	2	24	83
	21	Ethyl acetate (EtOAc)	2	48	42
	22	Acetonitrile (MeCN)	2	48	42
	23	4-Methylpentan-2-one (MIBK)	2	48	42
	24	Nitromethane ($MeNO_2$)	2	48	42
Nisoldipine (**3**)	25	Dimethyl sulfoxide (DMSO)	2	24	83
	26	Dimethylformamide (DMF)	2	12	167
	27	Methanol (MeOH)	2	48	42
	28	2,2,2-Trifluoroethanol (2,2,2-TFE)	2	24	83
	29	Toluene	2	96	21
	30	1,2-Dichloroethane (DCE)	2	24	83
	31	2-Methyltetrahydrofuran (2-MeTHF)	2	12	167
	32	1,4-Dioxane	2	12	167
	33	Ethyl acetate (EtOAc)	2	24	83
	34	Acetonitrile (MeCN)	2	24	83

	35	4-Methylpentan-2-one (MIBK)	2	48	42
	36	Nitromethane ($MeNO_2$)	2	48	42
Nitrendipine (**4**)	37	Dimethyl sulfoxide (DMSO)	2	24	83
	38	Dimethylformamide (DMF)	2	24	83
	39	Methanol (MeOH)	2	48	42
	40	2,2,2-Trifluoroethanol (2,2,2-TFE)	2	24	83
	41	Toluene	2	96	*Supernatant
	42	1,2-Dichloroethane (DCE)	2	48	42
	43	2-Methyltetrahydrofuran (2-MeTHF)	2	24	83
	44	1,4-Dioxane	2	24	83
	45	Ethyl acetate (EtOAc)	2	24	83
	46	Acetonitrile (MeCN)	2	48	42
	47	4-Methylpentan-2-one (MIBK)	2	48	42
	48	Nitromethane ($MeNO_2$)	2	48	42
Cilnidipine (**5**)	49	Dimethyl sulfoxide (DMSO)	2	24	83
	50	Dimethylformamide (DMF)	2	24	83
	51	Methanol (MeOH)	2	96	21
	52	2,2,2-Trifluoroethanol (2,2,2-TFE)	2	24	83
	53	Toluene	2	96	21
	54	1,2-Dichloroethane (DCE)	2	24	83
	55	2-Methyltetrahydrofuran (2-MeTHF)	2	24	83
	56	1,4-Dioxane	2	24	83
	57	Ethyl acetate (EtOAc)	2	24	83
	58	Acetonitrile (MeCN)	2	24	83
	59	4-Methylpentan-2-one (MIBK)	2	24	83
	60	Nitromethane ($MeNO_2$)	2	24	83
Nimodipine (**6**)	61	Dimethyl sulfoxide (DMSO)	2	24	83
	62	Dimethylformamide (DMF)	2	24	83
	63	Methanol (MeOH)	2	96	21
	64	2,2,2-Trifluoroethanol (2,2,2-TFE)	2	48	42
	65	Toluene	2	48	42
	66	1,2-Dichloroethane (DCE)	2	24	83
	67	2-Methyltetrahydrofuran (2-MeTHF)	2	24	83
	68	1,4-Dioxane	2	24	83
	69	Ethyl acetate (EtOAc)	2	24	83
	70	Acetonitrile (MeCN)	2	24	83
	71	4-Methylpentan-2-one (MIBK)	2	48	42
	72	Nitromethane ($MeNO_2$)	2	24	83

*Supernatant. Sample did not completely dissolve so the supernatant was taken for crystallisation experiments.

S4. Plate Set-Up

Using an SPT Labtech Mosquito Liquid-Handling Robot, 200 nL of one of four oils (PDMSO, FC-40, FY and mineral oil) were dispensed onto 96-well SWISSCI LCP plates with a 100-micron spacer. 50 nL of stock solution, containing the compound of interest, was collected from the parent plate and dispensed into the oil droplets within the wells (Figure S1). The plates were sealed with a glass cover slip and were stored in the dark at room temperature and after 2 weeks, evaluation of crystal growth was carried out visually by cross-polarised optical microscopy.

Vial	Volume of Oil		200 nL											
	Volume of Solvent		50 nL											
	Solvents		1	2	3	4	5	6	7	8	9	10	11	12
1	Solvent A	A	No oil	PDMSO					No oil	FY				
		B	No oil	FC-40					No oil	MO				
2	Solvent B	C	No oil	PDMSO					No oil	FY				
		D	No oil	FC-40					No oil	MO				
3	Solvent C	E	No oil	PDMSO					No oil	FY				
		F	No oil	FC-40					No oil	MO				
4	Solvent D	G	No oil	PDMSO					No oil	FY				
		H	No oil	FC-40					No oil	MO				

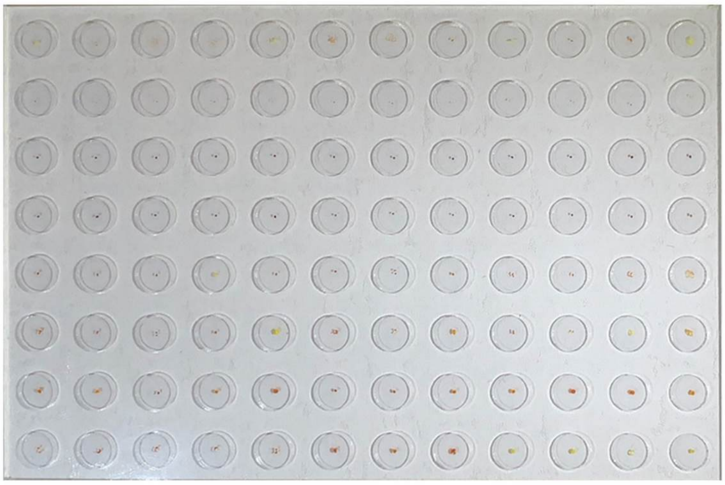

Figure S1 Top: example oil and solvent plate layout and bottom: image of a final plate.

S5. Plate Analysis

S5.1. End-Point Observations of ENaCt Experiments

After 14 days, the 96 well plates were examined by cross-polarised light microscopy, and the results of the ENaCt experiments in each well were classified as: F = fail (caused by a dispensing failure, resulting in no encapsulated droplet formation within the well); 1: remains in solution; 2: oiled-out or non-crystalline solid; 3: micro-crystalline solid; 4: crystals suitable for X-ray diffraction analysis (Figure S2).

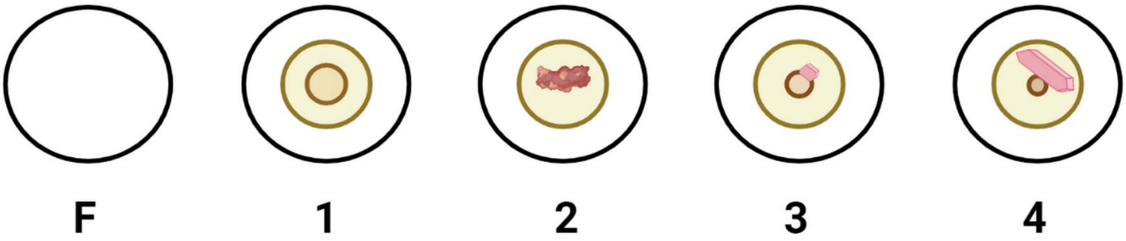

Figure S2 Top view of an ENaCt experiment where F = fail, 1 = sample remaining in solution, 2 = amorphous or oily material, 3 = microcrystalline sample and 4 = single crystal(s) suitable for SCXRD analysis.

S5.2. Plate readouts

**Felodipine_Plate 1**														
**Vial**	**Volume of Oil**		**200 nL**											
	**Volume of Solvent**		**50 nL**											
	**Solvents**		**1**	**2**	**3**	**4**	**5**	**6**	**7**	**8**	**9**	**10**	**11**	**12**
**1**	DMSO	**A**	2	2	2	2	2	2	2	2	2	2	2	2
	DMSO	**B**	2	2	2	4	2	2	2	2	4	4	4[a]	4
**2**	DMF	**C**	2	1	2	1	1	1	1	1	2	2	1	2
	DMF	**D**	2	2	1	1	2	4	2	4[b]	2	4	4	4
**3**	MeOH	**E**	2	2	2	2	2	2	2	4	3	2	2	2
	MeOH	**F**	2	3	3	3	2	4	1	4	4[a]	4[a]	2	4
**4**	2,2,2-TFE	**G**	2	2	2	2	2	2	2	2	2	2	2	2
	2,2,2-TFE	**H**	2	2	2	2	2	2	2	4	4	4	4[b]	4

**Felodipine_Plate 2**														
**Vial**	**Volume of Oil**		**200 nL**											
	**Volume of Solvent**		**50 nL**											
	**Solvents**		**1**	**2**	**3**	**4**	**5**	**6**	**7**	**8**	**9**	**10**	**11**	**12**
**5**	Toluene	**A**	3	3	2	3	3	2	2	2	2	2	2	2
	Toluene	**B**	2	2	2	4[a]	2	4	2	4	4[c]	4	4	4
**6**	DCE	**C**	1	2	2	2	2	2	2	4	2	2	2	2
	DCE	**D**	2	2	2	2	2	2	1	4	4	4	4[a]	4
**7**	2-MeTHF	**E**	4	2	2	2	2	2	3	2	2	2	2	2
	2-MeTHF	**F**	1	1	4	2	2	2	2	4[b]	4	4	4	4
**8**	1,4-Dioxane	**G**	2	2	2	2	2	2	2	2	2	2	2	2
	1,4-Dioxane	**H**	2	2	2	2	2	2	2	4	4[b]	4	4[b]	4

**Felodipine_Plate 3**														
**Vial**	**Volume of Oil**		**200 nL**											
	**Volume of Solvent**		**50 nL**											
	**Solvents**		**1**	**2**	**3**	**4**	**5**	**6**	**7**	**8**	**9**	**10**	**11**	**12**
**9**	EtOAc	**A**	2	2	2	2	2	2	2	2	2	2	2	2
	EtOAc	**B**	1	2	2	2	2	3	2	4	4[b]	4	4[d]	4
**10**	MeCN	**C**	1	2	2	2	2	2	2	2	2	2	2	2
	MeCN	**D**	2	2	2	2	2	2	2	4	4	4	4[b]	4
**11**	MIBK	**E**	2	2	3	2	2	2	2	2	2	2	2	2
	MIBK	**F**	2	2	2	2	2	2	2	4[b]	4	4	4	4
**12**	$MeNO_2$	**G**	2	2	2	2	2	2	3	4	2	1	2	1
	$MeNO_2$	**H**	2	4	4	4[a]	4	2	1	4	4	4	4[a]	4

Figure S3 Plate readouts for felodipine (**1**). [a] Unit cell data collected, matching CSD REFCODE: DONTIJ03 (form IV), [b] unit cell data collected, matching CSD REFCODE: DONTIJ (form I), [c] crystal selected for SCXRD analysis, and a full data collection completed (felodipine (**1**), 2263297), matching CSD REFCODE: DONTIJ03 (form IV) and [d] crystal selected for SCXRD analysis, and a full data collection completed (2295303), matching CSD REFCODE: DONTIJ (form I).

Nifidipine_Plate 1														
Vial	Volume of Oil		200 nL											
	Volume of Solvent		50 nL											
	Solvents		1	2	3	4	5	6	7	8	9	10	11	12
13	DMSO	A	2	2	2	2	2	2	2	3	4[a]	3	2	4
	DMSO	B	2	3	3	3	4[a]	4	2	2	2	2	2	4[a]
14	DMF	C	2	2	2	2	2	2	2	2	2	2	2	2
	DMF	D	2	4	4[b]	2	2	2	2	4	4	2	2	4[b]
15	MeOH	E	2	2	2	2	2	2	2	2	2	2	2	2
	MeOH	F	2	2	2	2	2	2	2	2	2	2	2	2
16	2,2,2-TFE	G	3	3	3	3	3	3	2	2	2	3	3	3
	2,2,2-TFE	H	3	3	3	3	3	3	2	4	4[c]	4	4	4[a]

Nifidipine_Plate 2														
Vial	Volume of Oil		200 nL											
	Volume of Solvent		50 nL											
	Solvents		1	2	3	4	5	6	7	8	9	10	11	12
17	Toluene	A	2	2	2	2	2	2	2	2	2	2	2	2
	Toluene	B	2	2	2	2	2	2	2	2	2	2	2	2
18	DCE	C	2	2	2	2	2	2	2	4[b]	3	3	3	2
	DCE	D	2	2	2	2	2	2	2	3	3	3	3	3
19	2-MeTHF	E	2	2	2	2	2	2	2	2	2	3	4[a]	2
	2-MeTHF	F	3	3	3	3	3	2	2	2	2	2	2	2
20	1,4-Dioxane	G	3	3	3	3	3	3	2	3	3	3	3	3
	1,4-Dioxane	H	2	3	3	3	3	3	2	4[e]	4	4[d]	4	4[e]

Nifidipine_Plate 3														
Vial	Volume of Oil		200 nL											
	Volume of Solvent		50 nL											
	Solvents		1	2	3	4	5	6	7	8	9	10	11	12
21	EtOAc	A	2	2	2	2	2	2	2	2	2	2	2	2
	EtOAc	B	2	3	3	2	2	2	2	3	3	3	3	3
22	MeCN	C	2	3	3	3	3	3	2	3	3	3	3	3
	MeCN	D	2	2	4[a]	2	2	2	2	2	2	2	3	4[a]
23	MIBK	E	3	3	3	3	2	3	2	2	2	2	2	2
	MIBK	F	3	3	3	3	4[a]	2	2	3	3	3	4[a]	4
24	$MeNO_2$	G	2	2	2	2	2	2	2	2	2	2	2	2
	$MeNO_2$	H	2	2	2	3	2	4[a]	2	3	4	4[a]	4	3

Figure S4 Plate readouts for nifedipine (**2**). [a] Unit cell data collected, matching CSD REFCODE: BICCIZ (α form), [b] unit cell data collected, matching CSD REFCODE: BICCIZ02 (β form), [c] crystal selected for SCXRD analysis, and a full data collection completed (nifedipine (**2**), 2263411) matching CSD REFCODE: BICCIZ06 (α form), [d] crystal selected for SCXRD analysis, and a full data collection completed (nifedipine•1,4-dioxane solvate (**2•1,4-dioxane**), 2263278), and [e] unit cell data collected, matching nifedipine•1,4-dioxane solvate (**2•1,4-dioxane**).

Nisoldipine_Plate 1														
Vial	Volume of Oil		200 nL											
	Volume of Solvent		50 nL											
	Solvents		1	2	3	4	5	6	7	8	9	10	11	12
25	DMSO	A	2	2	2	2	2	2	2	2	2	2	2	2
	DMSO	B	2	2	2	2	2	2	2	2	2	2	2	2
26	DMF	C	2	1	1	1	1	1	1	1	2	2	2	2
	DMF	D	2	2	2	2	2	2	2	1	2	2	2	2
27	MeOH	E	1	1	1	1	1	1	1	1	1	1	1	1
	MeOH	F	1	1	1	1	1	1	2	2	1	1	1	2
28	2,2,2-TFE	G	1	1	1	1	1	1	1	2	1	1	1	1
	2,2,2-TFE	H	2	2	2	2	2	2	2	2	2	2	2	2

Nisoldipine_Plate 2														
Vial	Volume of Oil		200 nL											
	Volume of Solvent		50 nL											
	Solvents		1	2	3	4	5	6	7	8	9	10	11	12
29	Toluene	A	3	3	1	1	1	1	1	1	1	1	1	1
	Toluene	B	1	1	1	1	1	1	1	1	1	3	1	1
30	DCE	C	1	2	2	2	2	2	2	1	2	1	1	1
	DCE	D	2	2	1	1	2	2	2	2	2	2	2	2
31	2-MeTHF	E	2	1	1	1	1	1	2	1	1	1	1	1
	2-MeTHF	F	2	1	1	1	1	1	2	1	1	1	1	1
32	1,4-Dioxane	G	2	1	1	1	1	1	2	1	1	1	1	2
	1,4-Dioxane	H	1	2	1	1	1	1	1	1	1	1	1	1

Nisoldipine_Plate 3														
Vial	Volume of Oil		200 nL											
	Volume of Solvent		50 nL											
	Solvents		1	2	3	4	5	6	7	8	9	10	11	12
33	EtOAc	A	2	2	2	2	2	2	2	2	2	2	2	2
	EtOAc	B	2	2	2	2	2	2	2	2	2	2	2	2
34	MeCN	C	2	2	2	2	2	2	2	2	2	2	2	2
	MeCN	D	2	1	1	1	1	1	1	1	2	1	1	1
35	MIBK	E	2	2	2	2	2	2	2	2	2	3	4[a]	4
	MIBK	F	2	2	2	2	2	2	2	3	3	3	3	2
36	$MeNO_2$	G	2	2	2	2	2	2	2	2	2	2	2	2
	$MeNO_2$	H	2	1	1	1	2	2	2	2	2	2	2	2

Figure S5 Plate readouts for nisoldipine (**3**). [a] Crystal selected for SCXRD analysis, and a full collection completed (nisoldipine (**3**), 2298790), matching CSD REFCODE: FULPAD.

Nitrendipine_Plate 1														
Vial	Volume of Oil		200 nL											
	Volume of Solvent		50 nL											
	Solvents		1	2	3	4	5	6	7	8	9	10	11	12
37	DMSO	A	4[a]	4	4	4[a]	4	4	4	4	4[a]	4	4	4
	DMSO	B	2	4	4	3	4[a]	4	3	4	4	4	2	4[a]
38	DMF	C	2	2	4	4	4	4	2	2	2	2	2	2
	DMF	D	4	4	4[a]	3	2	3	2	4[b]	3	4	4[a]	4
39	MeOH	E	2	2	2	2	2	3	2	2	2	2	2	2
	MeOH	F	2	4[a]	4	4[a]	4	3	2	3	3	3	3	2
40	2,2,2-TFE	G	3	3	4	4	3	4[a]	2	3	3	3	3	3
	2,2,2-TFE	H	2	2	3	4	3	3	2	4	4	4[a]	4	3

Nitrendipine_Plate 2														
Vial	Volume of Oil		200 nL											
	Volume of Solvent		50 nL											
	Solvents		1	2	3	4	5	6	7	8	9	10	11	12
41	Toluene	A	2	2	2	2	2	2	2	3	3	3	4[a]	3
	Toluene	B	2	3	3	3	3	3	2	3	3	3	3	3
42	DCE	C	2	3	3	3	3	4[a]	2	3	3	3	3	3
	DCE	D	2	3	3	3	3	3	2	3	3	3	3	3
43	2-MeTHF	E	2	3	3	3	3	3	3	3	3	3	3	3
	2-MeTHF	F	2	2	2	2	2	2	2	2	4[a]	2	2	2
44	1,4-Dioxane	G	2	3	3	3	3	3	2	3	3	3	3	4[a]
	1,4-Dioxane	H	2	3	3	3	3	3	2	3	3	3	3	4[a]

Nitrendipine_Plate 3														
Vial	Volume of Oil		200 nL											
	Volume of Solvent		50 nL											
	Solvents		1	2	3	4	5	6	7	8	9	10	11	12
45	EtOAc	A	2	4[a]	3	4[a]	3	3	2	3	3	3	2	2
	EtOAc	B	3	3	3	3	2	3	3	3	3	3	3	2
46	MeCN	C	2	3	3	4	4[a]	3	3	4[a]	3	4	4	2
	MeCN	D	2	3	3	3	3	3	2	3	3	3	3	3
47	MIBK	E	2	3	3	3	3	3	2	3	3	3	3	3
	MIBK	F	2	2	2	2	2	2	2	2	2	2	2	4
48	$MeNO_2$	G	2	3	3	3	3	3	2	3	3	2	3	3
	$MeNO_2$	H	2	3	4[a]	3	3	4	2	3	3	2	4[a]	3

Figure S6 Plate readouts for nitrendipine (**4**). [a] Unit cell data collected, matching CSD REFCODE: JEXKUS and [b] crystal selected for SCXRD analysis, and a full data collection completed (nitrendipine (**4**), 2215879), matching CSD REFCODE: JEXKUS.

**Cilnidipine_Plate 1**														
**Vial**	**Volume of Oil**		**200 nL**											
	**Volume of Solvent**		**50 nL**											
	**Solvents**		**1**	**2**	**3**	**4**	**5**	**6**	**7**	**8**	**9**	**10**	**11**	**12**
**49**	DMSO	**A**	1	1	1	1	1	1	1	1	1	1	1	1
	DMSO	**B**	1	1	1	1	1	1	1	1	1	1	1	1
**50**	DMF	**C**	1	1	1	1	1	1	1	1	1	1	1	1
	DMF	**D**	1	1	1	1	1	1	1	1	1	1	1	1
**51**	MeOH	**E**	1	1	2	2	2	1	1	1	2	1	1	2
	MeOH	**F**	1	2	2	2	1	2	1	1	2	2	1	1
**52**	2,2,2-TFE	**G**	1	1	1	1	2	2	1	2	2	2	1	2
	2,2,2-TFE	**H**	1	1	1	1	1	1	1	1	1	1	1	1

**Cilnidipine_Plate 2**														
**Vial**	**Volume of Oil**		**200 nL**											
	**Volume of Solvent**		**50 nL**											
	**Solvents**		**1**	**2**	**3**	**4**	**5**	**6**	**7**	**8**	**9**	**10**	**11**	**12**
**53**	Toluene	**A**	3	3	3	3	3	3	2	3	4[b]	4	4[a]	4
	Toluene	**B**	2	4[b]	3	3	2	2	2	3	3	3	3	3
**54**	DCE	**C**	1	2	2	2	2	2	1	1	2	2	1	1
	DCE	**D**	1	1	2	2	2	2	1	2	2	3	4[b]	3
**55**	2-MeTHF	**E**	1	2	2	2	2	2	1	2	1	1	1	1
	2-MeTHF	**F**	1	1	1	1	1	1	1	1	1	1	1	1
**56**	1,4-Dioxane	**G**	1	1	1	1	2	1	1	2	1	1	1	1
	1,4-Dioxane	**H**	1	1	2	2	1	2	1	1	2	1	1	2

**Cilnidipine_Plate 3**														
**Vial**	**Volume of Oil**		**200 nL**											
	**Volume of Solvent**		**50 nL**											
	**Solvents**		**1**	**2**	**3**	**4**	**5**	**6**	**7**	**8**	**9**	**10**	**11**	**12**
**57**	EtOAc	**A**	1	2	2	2	2	1	1	1	1	1	1	1
	EtOAc	**B**	1	1	1	1	2	1	1	1	2	2	2	2
**58**	MeCN	**C**	1	2	2	1	1	2	1	1	1	1	1	1
	MeCN	**D**	1	1	2	1	1	1	1	1	2	2	2	2
**59**	MIBK	**E**	1	2	2	2	2	2	2	2	1	2	2	2
	MIBK	**F**	1	1	1	1	1	1	1	1	1	1	2	1
**60**	[illegible]O_2	**G**	1	1	1	1	1	1	1	1	1	1	1	1
	[illegible]O_2	**H**	1	2	1	1	3	2	1	1	1	4[b]	1	1

Figure S7 Plate readouts for cilnidipine (**5**). [a] Crystal selected for SCXRD analysis, and a full data collection completed (cilnidipine (**5**), 2215828), matching CSD REFCODE: VELZUI. [b] Unit cell data collected, matching CSD REFCODE: VELZUI.

Nimodipine_Plate 1														
Vial	Volume of Oil		200 nL											
	Volume of Solvent		50 nL											
	Solvents		1	2	3	4	5	6	7	8	9	10	11	12
61	DMSO	A	4	4	4	4	4	4	2	4	4[a]	4	4	3
	DMSO	B	4	4	4[b]	4	4	4	2	4	4	4	4	4[b]
62	DMF	C	1	2	1	1	1	3	1	1	1	1	1	1
	DMF	D	1	2	1	2	1	1	2	1	1	1	1	1
63	MeOH	E	1	1	1	1	1	1	1	1	1	1	1	1
	MeOH	F	1	1	1	1	1	1	1	1	1	1	1	1
64	2,2,2-TFE	G	1	1	1	1	1	1	1	1	1	1	1	1
	2,2,2-TFE	H	1	1	1	1	1	1	1	1	1	1	1	1

Nimodipine_Plate 2														
Vial	Volume of Oil		200 nL											
	Volume of Solvent		50 nL											
	Solvents		1	2	3	4	5	6	7	8	9	10	11	12
65	Toluene	A	1	4	3	3	4[c]	4	4	2	2	2	2	2
	Toluene	B	2	2	2	3	3	3	2	4[c]	4	4	4	4
66	DCE	C	2	2	2	2	1	1	1	1	1	1	1	1
	DCE	D	2	2	2	2	2	3	3	1	1	1	1	1
67	2-MeTHF	E	1	1	2	1	1	1	2	1	1	2	2	2
	2-MeTHF	F	1	1	1	1	2	1	1	1	1	4[c]	1	1
68	1,4-Dioxane	G	1	1	1	1	1	1	1	1	1	1	1	1
	1,4-Dioxane	H	1	2	1	1	3	2	2	1	1	1	1	1

Nimodipine_Plate 3														
Vial	Volume of Oil		200 nL											
	Volume of Solvent		50 nL											
	Solvents		1	2	3	4	5	6	7	8	9	10	11	12
69	EtOAc	A	1	2	2	3	1	1	1	2	2	2	2	2
	EtOAc	B	1	1	1	1	2	1	1	1	1	1	4[c]	4
70	MeCN	C	2	1	1	1	2	2	1	1	1	1	1	1
	MeCN	D	1	1	3	1	1	1	1	1	1	1	4[c]	1
71	MIBK	E	2	2	2	2	2	2	2	2	2	2	2	2
	MIBK	F	2	2	2	2	2	2	2	2	2	2	2	4[c]
72	$MeNO_2$	G	1	1	2	1	2	2	2	2	2	2	2	2
	$MeNO_2$	H	1	2	2	2	1	2	1	1	4[d]	4	4[c]	4

Figure S8 Plate readouts for nimodipine (**6**). [a] Crystal selected for SCXRD analysis, and a full data collection completed (nimodipine•DMSO solvate (**6•DMSO**), 2215878). [b] Unit cell data collected, matching. Nimodipine•DMSO solvate (**6•DMSO**). [c] Unit cell data collected, matching CSD REFCODE: VAWWEW. [d] Crystal selected for SCXRD analysis, and a full data collection completed (nimodipine (**6**), 2263295), matching CSD REFCODE: VAWWEW.

S5.3. Three-Dimensional Plots of Crystallisation Outcomes from ENaCt

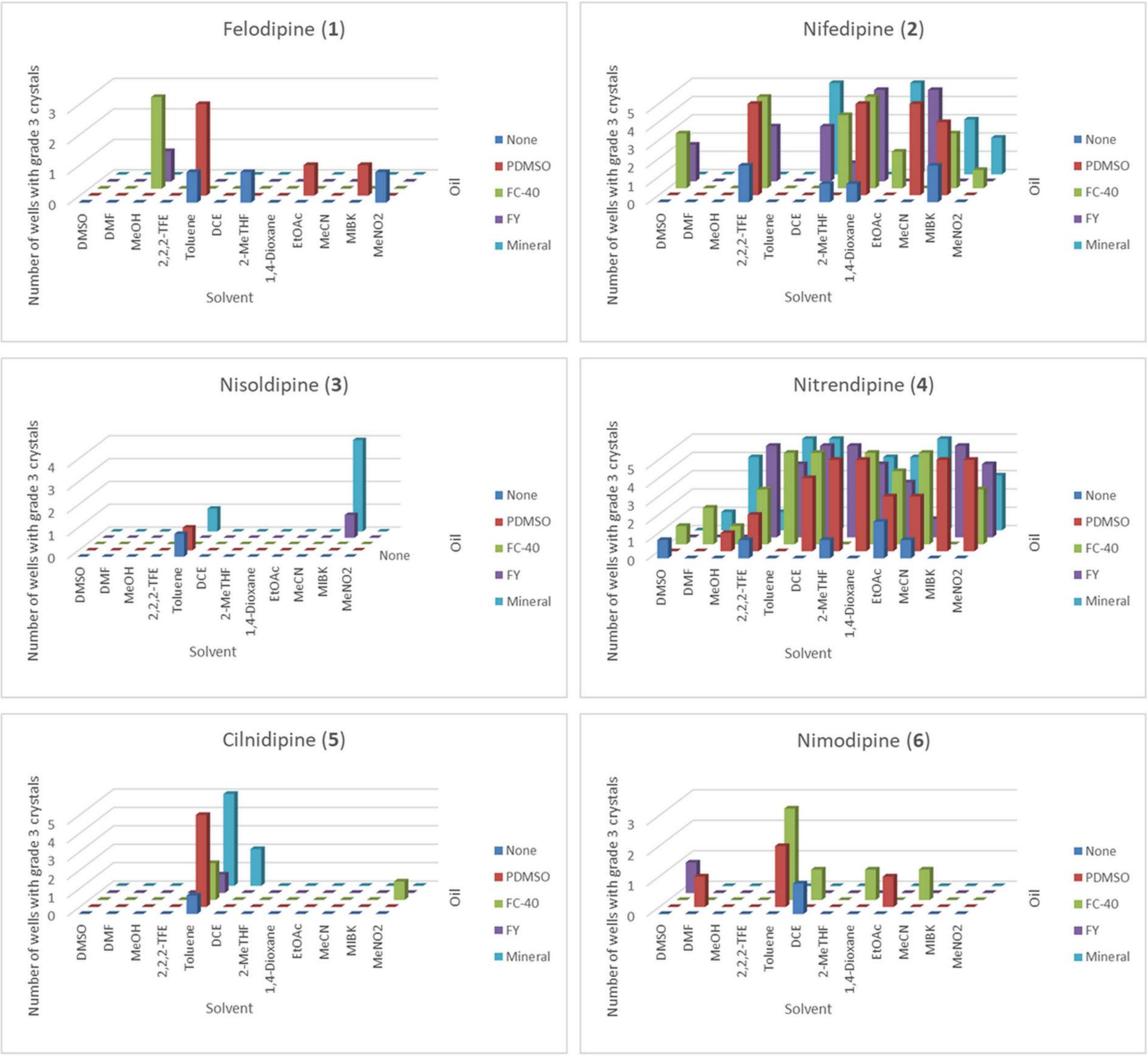

Figure S9 ENaCt conditions that provided crystals of grade 3 for dihydropyridine APIs.

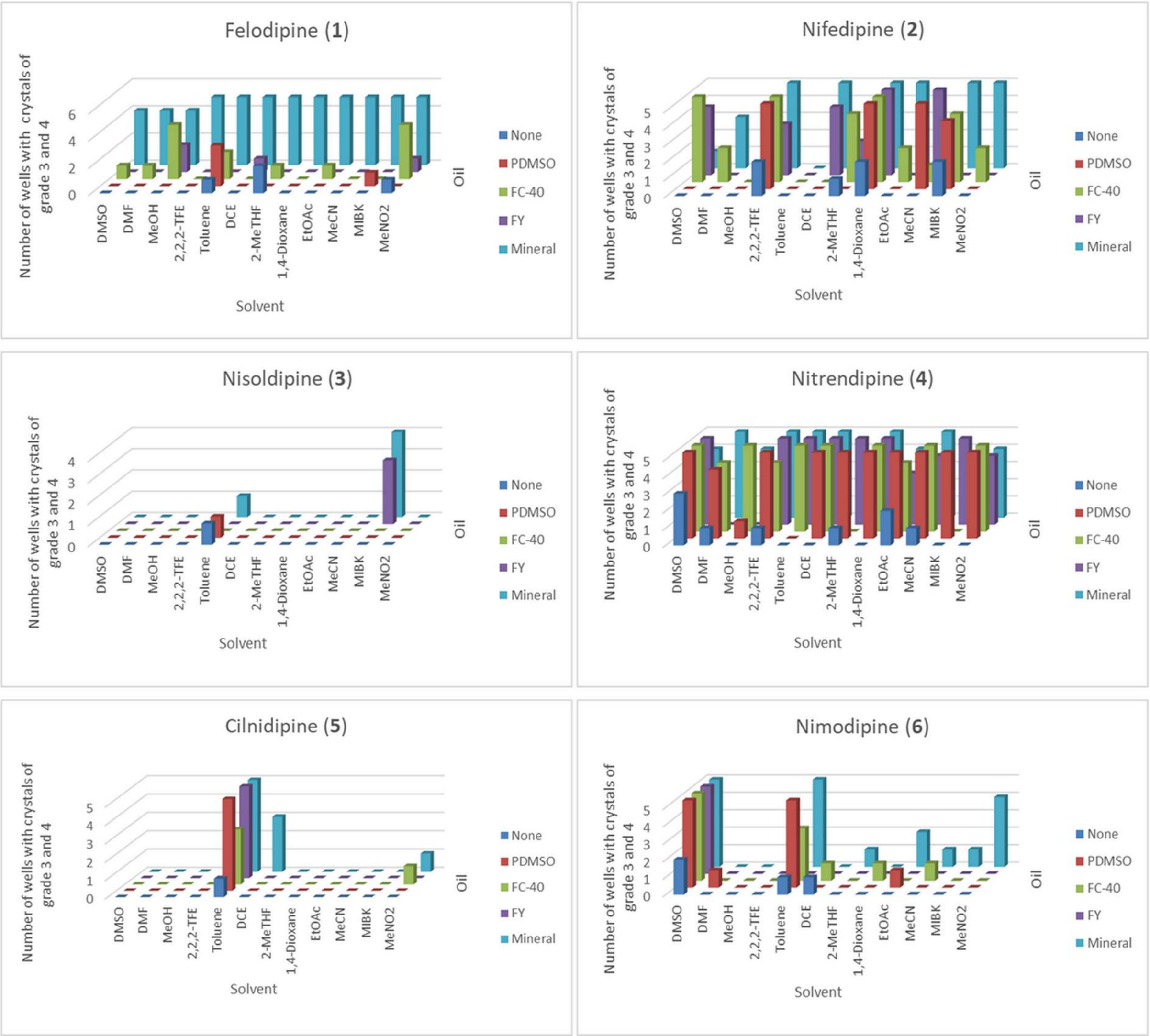

Figure S10 ENaCt conditions that provided crystals of grade 3 and 4 for dihydropyridine APIs.

S5.4. Crystals Selected for SCXRD

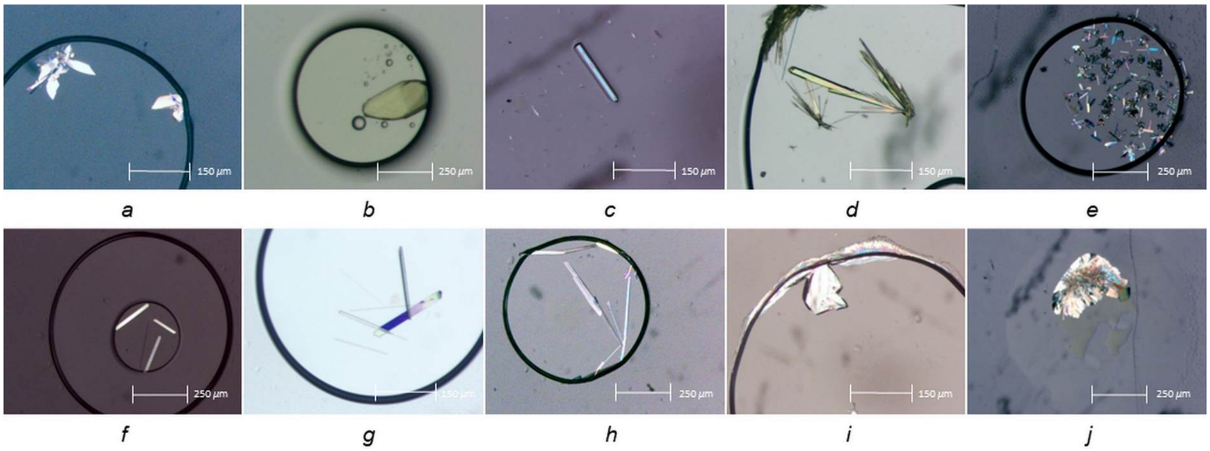

Figure S11 Plate-like crystals (form IV) were obtained from a solution of felodipine (**1**) in toluene with MO (P2_B9), (b) plate-like crystals (α form) were obtained from a solution of nifedipine (**2**) in 2,2,2-TFE with MO oil (P1_H9), (c) rod-like crystal obtained from a solution of nifedipine in 1,4-dioxane (**2•1,4-dioxane**) with MO oil (P2_H10), (d) lath-like crystals were obtained from a solution of nisoldipine (**3**) in MIBK with FY oil (P3_E11), (e) rod-like crystals were obtained from a solution of nitrendipine (**4**) in DMF with MO (P1_D8), (f) lath-like crystals were obtained from a solution of cilnidipine (**5**) in toluene with FY oil (P2_A11), (g) lath-like crystals were obtained from a solution of nimodipine (**6**) in $MeNO_2$ with MO (P3_H9), (h) lath-like crystals were obtained from a solution of nimodipine in DMSO (**6•DMSO**) with FY oil (P1_A9), (i) plate-like crystals (form I) were obtained from a solution of felodipine (**1**) in EtOAc with MO (P3_B11) and (j) plate-like crystals (β form) were obtained from a solution of nifedipine (**2**) in DCE with FY oil (P2_C8).
